# Pediatric Brain Tumors: A Case Report on the Rare Occurrence of Anaplastic Meningioma in a Young Child

**DOI:** 10.7759/cureus.82886

**Published:** 2025-04-24

**Authors:** Baderddine Mohammadine, Oualid Hmamouche, Benzagmout Mohammed, Chakour Khalid, Chaoui Faiz Mohammed

**Affiliations:** 1 Department of Neurosurgery, Center Hospitalier Universitaire (CHU) Hassan II, Fez, MAR; 2 Department of Neurosurgery, University Hospital Hassan II, Fez, MAR; 3 Department of Neurosurgery, Hassan II University Hospital of Fez, Fez, MAR

**Keywords:** anaplastic meningioma, multidisciplinary approach (mdt), pediatric brain tumors, pediatric meningioma, who grade 3

## Abstract

Meningiomas are uncommon in the pediatric population and often exhibit distinct characteristics compared to those in adult cases. We report the case of a five-year-old girl who presented with progressive right-sided exophthalmos, headaches, and seizures. Imaging revealed a large extra-axial mass in the right temporal region, extending into the cavernous sinus and orbital cavity. A subtotal surgical resection was performed, and histological examination confirmed an anaplastic meningioma, World Health Organization grade 3. The patient was referred for adjuvant radiotherapy. This case highlights the importance of recognizing atypical presentations in children and underscores the need for a multidisciplinary management approach in pediatric high-grade meningiomas.

## Introduction

Meningiomas are the most common primary intracranial tumors (38.1%) [1]. Pediatric meningiomas represent approximately 1%-2% of primary intracranial tumors in individuals under the age of 21. The mean age at diagnosis is 12 years (±4 years). Congenital and infantile cases are extremely rare [2]. Their epidemiological, histopathological, and radiological characteristics differ from those observed in adults.

## Case presentation

**Figure 1:**
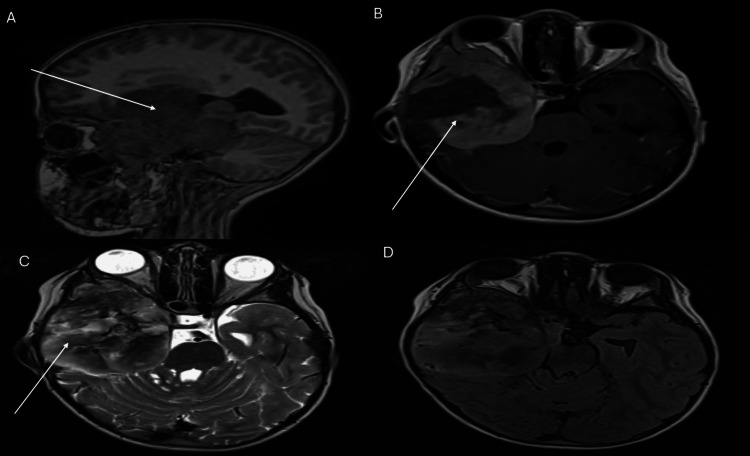
MRI brain imaging. A voluminous right temporal lesion with an extra-axial appearance is observed, showing low signal on T1 (A), high signal on T2 (C), and heterogeneous enhancement following contrast injection (B). Isointense on FLAIR sequence (D). The mass measures 60 mm in transverse diameter and 78 mm in anteroposterior diameter. It occupies the entire right temporal fossa, extending into the cavernous sinus medially, with a mass effect on the midbrain posteriorly and the third ventricle superiorly. Anteriorly, the mass invades the posterior part of the ocular globe, causing exophthalmos MRI: magnetic resonance imaging; FLAIR: fluid-attenuated inversion recovery

A five-year-old girl presented with right-sided exophthalmos associated with headaches and a seizure episode, evolving over the past two weeks before admission. Neurological examination revealed a conscious patient with left-sided hemiparesis, sufficient for walking. Ophthalmological examination revealed right nonpulsatile exophthalmos, without signs of inflammation or lacrimation, papilledema, and bilateral visual acuity reduction. Brain imaging showed a sizeable tumoral process measuring 60 mm in transverse diameter and 78 mm in anteroposterior diameter, with extra-axial characteristics occupying the right temporal fossa, extending superiorly to the frontal region, medially to the cavernous sinus, and anteriorly penetrating the ipsilateral ocular globe (Figure 1). The patient underwent surgery and the resection was subtotal (SR) because the tumor adhered strongly to the cavernous sinus, and the histopathological study confirmed an anaplastic meningioma, World Health Organization (WHO) grade 3. The patient was subsequently referred to the radiotherapy department.

## Discussion

Global data on the incidence of pediatric meningiomas are quite limited. In the Netherlands, pediatric meningiomas occur at an average rate of one case per one million children annually [3].

The clinical and epidemiological characteristics of pediatric meningiomas differ from those in adults. In the most recent literature review, Tauziède-Espariat et al. [4] report that, unlike in adults, where meningiomas predominantly affect women, pediatric meningiomas occur equally in both men and women (52% vs. 48% of reported cases). Pediatric meningiomas can occur throughout the central nervous system (CNS), with the most common sites being the cerebral convexities (46% of cases) and the skull base (27% of cases). In children, there is a higher incidence of intraventricular and spinal meningiomas, representing 10% and 7% of cases, respectively. Other locations include the posterior fossa (7% of cases) and, less commonly, the orbit, optic nerve, and olfactory grooves, which account for less than 4% of reported cases. Meningiomatosis is observed in 15% of pediatric cases. The presenting symptoms vary depending on the tumor's location. Given their preference for supratentorial areas, pediatric meningiomas often present with seizures and symptoms of intracranial hypertension [4]. Pediatric meningiomas are more frequently associated with genetic syndromes, such as neurofibromatosis type II (30%) [5].

MRI is the radiological imaging modality of choice. Children meningiomas are usually superficial, appearing as isointense or hypointense masses on T1-weighted scans, and hyperintense on T2-weighted MRI [6].

Currently, in the World Health Organization Central Nervous System fifth edition (WHO CNS5) classification, meningiomas are defined as a single tumor entity comprising 15 histopathological subtypes, with malignancy grading now applied intratumorally and independently of the specific subtype [2,7]. Therefore, the WHO CNS5 classification supports the use of molecular biomarkers to refine tumor classification and malignancy grading; however, their application is not mandatory for diagnosis when definitive histopathological identification of a meningioma subtype is available [2,7,8]. There are three grades of malignancy (grades 1-3) based on the subtype and histopathology (Table 1) [2,7].

**Table 1:** WHO 2021 histopathological classification (meningioma subtypes classification) TERT: telomerase reverse transcriptase; WHO: World Health Organization Source: [2,7]

WHO grade	Histological type	Criteria
1/2	Meningothelial meningioma, fibrous meningioma, transitional meningioma, psammomatous meningioma, angiomatous meningioma, microcystic meningioma, secretory meningioma, and lymphoplasmacyte-rich meningioma	-
2	Atypical meningioma (including brain-infiltrative meningiomas), chordoid meningioma, and clear cell meningioma	19 mitoses within 10 consecutive fields (0.16 mm²), brain infiltration, or at least three of the following criteria: increased cellularity, small cells with a high nucleus-to-cytoplasm ratio, prominent nucleoli sheet-like growth, and focal spontaneous necrosis
3	Anaplastic (malignant) meningioma	More than 20 mitoses in 10 consecutive fields, frank anaplasia, TERT mutation, or homozygous deletion of CDKN2A and/or CDKN2B

The frequency of high-grade meningiomas is higher in children [9,10]. Pediatric meningiomas exhibit a more aggressive biological behavior compared to those in adults, with a significant proportion of patients experiencing disease progression following surgery [11].

Due to their rarity, pediatric meningiomas are often treated according to adult protocols, which may be inappropriate, as meningiomas in children have distinct biological and clinical features. Treatment primarily involves surgical resection, but decision-making regarding adjuvant treatment, particularly radiotherapy, must be cautious, considering the risk of long-term toxicity on the developing brain vs. the risk of multiple recurrences. A multidisciplinary approach (multidisciplinary team) in pediatric neuro-oncology is essential to tailor treatment to each patient [6]. Gross total resection (GTR) is the preferred treatment for symptomatic meningiomas. In contrast, radiotherapy remains the sole available adjuvant therapy and may be required for tumors that cannot be fully excised. Anaplastic grade III meningiomas have a high likelihood of recurrence, irrespective of resection status. However, this risk is lower following GTR. Therefore, adjuvant radiotherapy should be considered at the time of primary diagnosis, regardless of the surgical outcome [12].

## Conclusions

Pediatric meningiomas, especially anaplastic grade III tumors, are rare and aggressive, with a high risk of recurrence even after surgical resection. Due to their distinct biological characteristics, management should be tailored to each patient, combining GTR when possible with adjuvant radiotherapy for incomplete resections or high-grade tumors. A multidisciplinary approach is essential to optimize treatment outcomes and minimize long-term risks to the developing brain.
